# Multisite randomised controlled trial of trauma-focused cognitive behaviour therapy for psychosis to reduce post-traumatic stress symptoms in people with co-morbid post-traumatic stress disorder and psychosis, compared to treatment as usual: study protocol for the STAR (Study of Trauma And Recovery) trial

**DOI:** 10.1186/s13063-022-06215-x

**Published:** 2022-05-23

**Authors:** Emmanuelle Peters, Amy Hardy, Robert Dudley, Filippo Varese, Kathryn Greenwood, Craig Steel, Richard Emsley, Nadine Keen, Samantha Bowe, Sarah Swan, Raphael Underwood, Eleanor Longden, Sarah Byford, Laura Potts, Margaret Heslin, Nick Grey, Doug Turkington, David Fowler, Elizabeth Kuipers, Anthony Morrison

**Affiliations:** 1 Department of Psychology, Institute of Psychiatry, Psychology & Neuroscience, King’s College London, London, UK; 2 South London & Maudsley NHS Foundation Trust, London, UK; 3 Cumbria, Northumberland, Tyne and Wear NHS Foundation Trust, Newcastle upon Tyne, UK; 4 Newcastle University, London, UK; 5 School of Health Sciences, University of Manchester, Manchester Academic Health Science Centre, Manchester, UK; 6 Complex Trauma and Resilience Research Unit, Greater Manchester Mental Health NHS Foundation Trust, Manchester Academic Health Science Centre, Manchester, UK; 7 Research and Development, Sussex Partnership NHS Foundation Trust, Brighton, UK; 8 School of Psychology, University of Sussex, London, UK; 9 Oxford Centre for Psychological Health, Oxford Health NHS Foundation Trust, Oxford, UK; 10 Oxford Institute of Clinical Psychology Training and Research, University of Oxford, Oxford, UK; 11 Department of Biostatistics and Health Informatics, Institute of Psychiatry, Psychology & Neuroscience, King’s College London, London, UK; 12 Psychosis Research Unit, Greater Manchester Mental Health NHS Foundation Trust, Manchester Academic Health Science Centre, Manchester, UK; 13 Health Service & Population Research, Institute of Psychiatry, Psychology & Neuroscience, King’s College London, London, UK

**Keywords:** Post-traumatic stress disorder (PTSD), Psychosis, Schizophrenia-spectrum disorder, Trauma, Cognitive behaviour therapy, Trauma-focused therapy, Trauma memory reprocessing, Delusions, Hallucinations

## Abstract

**Background:**

People with psychosis have high rates of trauma, with a post-traumatic stress disorder (PTSD) prevalence rate of approximately 15%, which exacerbates psychotic symptoms such as delusions and hallucinations. Pilot studies have shown that trauma-focused (TF) psychological therapies can be safe and effective in such individuals. This trial, the largest to date, will evaluate the clinical effectiveness of a TF therapy integrated with cognitive behaviour therapy for psychosis (TF-CBTp) on post-traumatic stress symptoms in people with psychosis. The secondary aims are to compare groups on cost-effectiveness; ascertain whether TF-CBTp impacts on a range of other meaningful outcomes; determine whether therapy effects endure; and determine acceptability of the therapy in participants and therapists.

**Methods:**

Rater-blind, parallel arm, pragmatic randomised controlled trial comparing TF-CBTp + treatment as usual (TAU) to TAU only. Adults (*N* = 300) with distressing post-traumatic stress and psychosis symptoms from five mental health Trusts (60 per site) will be randomised to the two groups. Therapy will be manualised, lasting 9 months (m) with trained therapists. We will assess PTSD symptom severity (primary outcome); percentage who show loss of PTSD diagnosis and clinically significant change; psychosis symptoms; emotional well-being; substance use; suicidal ideation; psychological recovery; social functioning; health-related quality of life; service use, a total of four times: before randomisation; 4 m (mid-therapy); 9 m (end of therapy; primary end point); 24 m (15 m after end of therapy) post-randomisation. Four 3-monthly phone calls will be made between 9 m and 24 m assessment points, to collect service use over the previous 3 months. Therapy acceptability will be assessed through qualitative interviews with participants (*N* = 35) and therapists (*N* = 5–10). An internal pilot will ensure integrity of trial recruitment and outcome data, as well as therapy protocol safety and adherence. Data will be analysed following intention-to-treat principles using generalised linear mixed models and reported according to Consolidated Standards of Reporting Trials-Social and Psychological Interventions Statement.

**Discussion:**

The proposed intervention has the potential to provide significant patient benefit in terms of reductions in distressing symptoms of post-traumatic stress, psychosis, and emotional problems; enable clinicians to implement trauma-focused therapy confidently in this population; and be cost-effective compared to TAU through reduced service use.

**Trial registration:**

ISRCTN93382525 (03/08/20)

**Supplementary Information:**

The online version contains supplementary material available at 10.1186/s13063-022-06215-x.

## Administrative information

Note: the numbers in curly brackets in this protocol refer to SPIRIT checklist item numbers. The order of the items has been modified to group similar items (see http://www.equator-network.org/reporting-guidelines/spirit-2013-statement-defining-standard-protocol-items-for-clinical-trials/).

**Table:** 

Title {1}	Multisite Randomised Controlled Trial of Trauma-Focused Cognitive Behaviour Therapy for psychosis to reduce post-traumatic stress symptoms in people with co-morbid post-traumatic stress disorder and psychosis, compared to Treatment As Usual: Study protocol for the STAR (Study of Trauma And Recovery) trial
Trial registration {2}	ISRCTN registry, reference: ISRCTN93382525, registered on 03/08/20
Protocol version {3}	Version 3.02, 21.01.22
Funding {4}	This trial is funded by an NHS National Institute for Health Research (NIHR) Health Technology Assessment (HTA), NIHR128623.
Author details {5a}	King’s College London, Institute of Psychiatry, Psychology & Neuroscience, Psychology Department South London and Maudsley NHS Foundation Trust *EP, AH, NK, SS, RU, EK* King’s College London, Institute of Psychiatry, Psychology & Neuroscience, Department of Biostatistics and Health Informatics *RE, LP* King’s College London, Institute of Psychiatry, Psychology & Neuroscience, Health Service & Population Research *SBy, MH* Cumbria, Northumberland, Tyne and Wear NHS Foundation Trust Newcastle University *RD, DT* School of Health Sciences, The University of Manchester, Manchester Academic Health Science Centre *FV, TM* Psychosis Research Unit, Greater Manchester Mental Health NHS Foundation Trust, Manchester Academic Health Science Centre *SBo, EL, TM* Complex Trauma and Resilience Research Unit, Greater Manchester Mental Health NHS Foundation Trust, Manchester Academic Health Science Centre *FV EL* Research and Development, Sussex Partnership NHS Foundation Trust School of Psychology, University of Sussex *KG, DF, NG* Oxford Centre for Psychological Health, Oxford Health NHS Foundation Trust University of Oxford *CS*
Name and contact information for the trial sponsor {5b}	Co-Sponsor: King’s College London Professor Reza Razavi, Room 5.31, James Clerk Maxwell Building, 57 Waterloo Road, London, SE1 8WA. 02078483224 reza.razavi@kcl.ac.uk Co-Sponsor: South London & Maudsley NHS Trust R&D Department, Room W1.08 Institute of Psychiatry, Psychology & Neuroscience (IoPPN) De Crespigny Park, London, SE5 8AF. 02078480339 slam-ioppn.research@kcl.ac.uk
Role of sponsor {5c}	This trial was designed by the research team in response to a commissioned call from the Funder (NIHR), who has also approved the content of the final research protocol. Neither the Funder nor the Co-Sponsors will have a role in data collection, management, analysis, or interpretation; nor in the writing of the final report or decision to submit the report. The views expressed in this publication are those of the authors and not necessarily those of the NHS, the NIHR or the Department of Health and Social Care.

## Introduction

### Background and rationale {6a}

People with psychosis report high rates of adversity and trauma, particularly interpersonal victimisation (e.g. emotional, physical, and sexual abuse/assaults) both in childhood and adulthood, with the majority having experienced multiple traumas (75–98% of those reporting trauma [1]). The prevalence rate of post-traumatic stress disorder (PTSD) in this population is approximately 15%, which is up to five times the general population rates [2]. PTSD is characterised by intrusive memories of the trauma, such as ‘flashbacks’, hyperarousal, and avoidance of trauma reminders. Post-traumatic symptoms are frequently intertwined with psychotic symptoms, such as delusions and hallucinations [3, 4]. However, in clinical practice PTSD is overlooked in many people with psychosis [2]. A single diagnosis often means that the psychosis is treated pharmacologically, but not the psychological effects of traumatic events. Such individuals have a poorer response to antipsychotic medication [5], and increased substance-abuse, self-harm, suicide behaviour, and psychiatric and medical hospitalisation than those with psychosis alone [1].

Cognitive behavioural therapy for psychosis (CBTp) is recommended for people with psychosis, as an adjunctive therapy to medication [6]. CBTp is a ‘trauma-informed’ therapy, in that it involves making sense of how trauma has shaped a person’s difficulties, and learning strategies for managing trauma-related distress [7, 8]. However, it does not focus directly on the key psychological mechanism in the development and maintenance of PTSD—vivid, sensory trauma memories that are poorly contextualised in autobiographical memory [4, 9, 10]. Trauma-focused CBT (TF-CBT) is recommended for PTSD [11], which includes ‘trauma memory reprocessing’, i.e. targeting trauma memories directly, through imaginal exposure, in vivo exposure, and experiential and cognitive techniques to modify their associated meanings. These techniques elaborate and contextualise trauma memories in autobiographical memory so that they become less distressing and less likely to intrude involuntarily (e.g. as flashbacks or nightmares). However, therapists can be reluctant to address PTSD symptoms directly in people with psychosis as they fear the memory reprocessing procedures may exacerbate psychotic symptoms [12]. These concerns have excluded people displaying psychotic symptoms from all prominent PTSD trials [13].

Three systematic reviews [14–16] have all concluded there is emerging evidence from open and pilot randomised controlled trials (RCTs) [17–19] and case-series studies [20] that treating PTSD can be safe and efficacious in psychosis. The largest RCT was carried out in The Netherlands [19], and recruited adults with a lifetime diagnosis of psychosis and meeting full diagnostic criteria for PTSD. Compared to the waiting list group, trauma-focused therapies led to improvements in PTSD symptoms with large between-group Cohen’s *d* [21] effect sizes (ES) (0.78; *p* < 0.001, in the Prolonged Exposure arm; 0.65; *p* = 0.001, in the Eye Movement Desensitization and Reprocessing (EMDR) arm) as assessed with a continuous measure of PTSD symptoms (Clinician-Administered PTSD Scale (CAPS) [22]). Furthermore, 57% in the Prolonged Exposure group (*N* = 53), and 60% in the EMDR condition (*N* = 55), achieved a loss of PTSD diagnosis, compared to 28% of the waiting list group (*N* = 47). End of therapy effects were maintained at both 6 m [19] and 12 m [23] follow-up time points, with similar results obtained on secondary outcomes [23, 24]. However, the 12-m follow-up analyses were within group only (comparing the 6 to 12 m outcomes), and further research is needed to ascertain between-group long-term effects.

There have been four recent UK studies (one RCT and three case-series studies) in this area [20, 25–27]. Steel and colleagues [25] also showed that psychological therapy was safe and feasible in a small RCT with people diagnosed with schizophrenia-spectrum disorders (*N* = 61). However, no difference was found between therapy and treatment-as-usual (TAU) groups on PTSD symptoms on the CAPS-S (CAPS for Schizophrenia [28]; either at the 6 m (end of therapy; ES = 0.26; *p* = 0.39) or 12 m (ES = 0.29; *p* = 0.39) follow-up time points, with both groups improving. There are two potential reasons for the discrepant results between the UK and Dutch trials. First, the therapy protocol in the Steel et al. trial involved cognitive restructuring only, without the exposure element, unlike the Dutch study. Second, participants did not meet full PTSD diagnostic criteria in the UK trial. As a result, participants presented with less severe, and potentially less stable, PTSD symptoms compared to other trials, potentially leading to some degree of spontaneous recovery occurring in both arms.

The proposed trial will address the limitations of the previous trials in several ways. First, our proposed intervention, TF-CBTp, includes trauma memory exposure, which is hypothesised to be central to effective trauma-focused therapy for PTSD [29]. This standard PTSD therapy will be integrated with the standard therapy for psychosis, CBTp, according to our previous theoretical models [3, 30, 31], practice recommendations [8] and case-series of TF-CBTp [20, 26, 27]. Second, all participants will meet PTSD diagnostic criteria. They will also be screened for the presence of at least one re-experiencing symptom [32, 33], to ensure specificity of presenting symptoms to PTSD [34], and on which to anchor the trauma memory reprocessing therapeutic procedures. The diagnostic interview will put particular emphasis on assessing symptom stability, i.e. continuous presence of symptoms, attributable to the index trauma, for 1 month minimum, as specified in the Diagnostic and Statistical Manual of Mental Disorders 5th Edition (DSM-5 [35]). Third, this study will differ from the Dutch trials (including a new, ongoing trial comparing Prolonged Exposure, EMDR, Cognitive Restructuring and Waiting List) [36] by only including participants with current distressing psychotic symptoms, rather than merely a lifetime psychotic disorder diagnosis. Last, between-group analyses 2 years post-randomisation will be reported to ascertain the sustainability of treatment effects.

Integrating TF therapy with CBTp means that it is more intensive and lengthier than the Prolonged Exposure intervention reported by the Dutch group (9 months compared to eight weekly 90-min sessions over 10 weeks, respectively). However, van den Berg and colleagues have since reported their protocol had too few sessions and have recommended longer therapy [37]. Psychosis and PTSD symptoms are often intertwined [3, 4], for example hearing the voice of an abuser, experiencing physical sensations of being interfered with, or visions of past torturers (i.e. auditory, somatic, and visual hallucinations); or believing past abusers implanted a chip in your brain to track you (paranoid delusions). In practice, it therefore makes little clinical sense to treat the PTSD and psychosis symptoms separately. The National Institute for Health and Care Excellence (NICE) [6] recommend a minimum of 16 sessions over 6 months or longer for psychosis, with more sessions leading to better outcomes [38]. Therapy lasting 12 months plus boosters is recommended for complex PTSD where there are multiple or chronic traumas [39], which is the case for most of the people with psychosis seen in services. Therefore, TF-CBTp is shorter and potentially less costly than if the two conditions were addressed separately. In general, integration of exposure procedures within standard therapies is preferable for complex, co-morbid populations [40, 41].

To conclude, the current evidence derives from a range of diverse small trials in different health settings. They clearly demonstrate feasibility and promise of useful effects, but a pragmatic effectiveness trial is now needed. NICE have recommended that ‘an adequately powered, multi-centre RCT is needed to test whether a CBT-based trauma reprocessing intervention can reduce PTSD symptoms and related distress in people with psychosis and schizophrenia’ [6]. The recent Cochrane Review [14] also recommended that good quality evidence is required on trauma-focused therapy in psychosis individuals with co-morbid PTSD. This study follows these recommendations and will test of whether TF-CBTp is safe, and clinically and cost-effective in people with psychosis.

#### Covid-19 adaptations to the trial

Adaptations were made to the protocol during the pandemic to enable assessments and the intervention to be delivered remotely either by videoconferencing or telephone, when in-person meetings were not possible. Although the drivers for these adaptations were compliance with UK government guidelines, there is evidence about the efficacy of remote delivery of PTSD psychological treatments dating back to more than a decade, both in terms of telehealth (see [42] for a review) and e-Mental Health (internet) interventions more generally (39 studies identified by a systematic review by [43]). There is evidence of non-inferiority from trials comparing remote with in-person delivery of treatment directly [44–46], in terms of PTSD symptom reductions and maintenance of treatment gains. The use of remote therapy does not affect therapy competence, adherence, and general fidelity [44, 47, 48]. Overall, a recent review [49] concluded that telehealth interventions for PTSD have a demonstrated evidence base of feasibility, acceptability, and comparable outcomes to traditional in-person therapy, without compromising the therapeutic process.

There is less research on telephone delivery, but several studies support the efficacy of telephone treatments for depression and anxiety [50, 51]. A review [52] found few differences in patient satisfaction in 15 studies comparing video conferencing or telephone therapy to in-person therapy. Of note, although there is a dearth of studies investigating videoconferencing in psychosis populations [53], in clinical practice it is the norm to have at least some therapy sessions delivered by phone with such populations, which reduces the number of missed sessions for this group who may have difficulty attending in person.

There has been some suggestions that remote delivery may adversely impact the therapeutic relationship, with some studies [46, 54] but not others [55] finding a slightly higher drop-out rate than in in-person PTSD treatments. To minimise this possibility, we incorporated the extensive guidelines that have been published for successful delivery of remote therapy in our therapy protocol and training [42, 56, 57]; see also resources at https://oxcadatresources.com/covid-19-resources/). In addition, our therapy protocol already includes digital materials (around providing psychoeducation and promoting control), which will benefit digital platforms and will facilitate therapy engagement.

We have included a measure of therapeutic alliance for participants in the therapy group that will allow us to monitor the potential impact of different modes of delivery on the therapeutic relationship. We will record the type of delivery used for each assessment and therapy session, which will enable us, if required, to do post hoc analyses comparing the use of different platforms on outcomes.

If appropriate, the adaptations will be deliverable for the entirety of the study, rather than just during the pandemic. Pre-pandemic, the drivers for developing remote treatments were to increase access to therapy when in-person meetings were not feasible [49]. Unsurprisingly, during 2020, there has been a step change in the implementation of these methods across the UK (and internationally). It is likely that some of these changes will remain post-pandemic, and we will be able to continue to offer remote delivery throughout the trial for those who prefer it. There is limited research evidence on patient preferences, but one study found that up to 50% of participants preferred remote delivery [46].

Equally, we know that not everyone is able or willing to access remote therapy, with older and disadvantaged groups being less likely to have access to the technology, including having the necessary digital literacy, and/or suitable private space [42, 58]. With regard to psychosis populations specifically, a recent study in one of the trial sites showed that 30% of people on a psychological therapy waiting list declined the offer of remote therapy [59]. Therefore, to adhere to patient choice, there will be the opportunity to carry out in-person therapy for those who choose to do so, even during Covid-19 restrictions, adhering to Trust policies and government guidelines to ensure safety of therapists and participants.

Overall, the current changes taking place throughout the UK and elsewhere will likely change the landscape of therapy delivery well beyond this pandemic [42] and will contribute to increased patient choice and access to therapy, especially for difficult to reach populations [58]. Delivering at least some therapy sessions by phone is already standard practice with psychosis population; going forward, there will be increased flexibility of delivery platforms to cater to different clinical needs and preferences, both across patients and within the duration of therapy for individual patients. We are therefore confident that our results will be valid and generalisable post-pandemic. We will also have access to further data on patient and therapist experience through our qualitative interviews, which will provide useful information about the effective use of the different options.

### Objectives {7}

Our research question is the following: Is TF-CBTp in addition to TAU clinically and cost-effective in reducing the severity of post-traumatic stress symptoms in people with PTSD and psychosis at the end of therapy, compared to TAU alone?

#### Primary aim

The primary aim is to evaluate the effectiveness of a manualised trauma-focused therapy for psychosis (TF-CBTp) on post-traumatic stress symptom severity in people with current PTSD and psychosis at the end of therapy (9 m post-randomisation).

#### Secondary aims

Secondary aims are as follows:

1. To compare the two groups at 9 m post-randomisation (end of therapy) on (i) percentage of individuals achieving a loss of PTSD diagnosis, and showing clinically significant change; (ii) PTSD symptom clusters; psychosis symptoms and associated distress; emotional well-being; suicidal ideation; substance use; psychological recovery; social functioning; (iii) cost-effectiveness;
2. To determine whether therapy effects endure 24 m post-randomisation (15 m post end of therapy), including both clinical and cost-effectiveness;
3. To determine the acceptability of TF-CBTp in participants and therapists.

### Trial design {8}

The STAR (Study of Trauma And Recovery) trial is a rater-blind, parallel arm RCT comparing an integrated therapy to address post-traumatic stress and psychosis symptoms (TF-CBTp) in addition to TAU, to TAU alone, in individuals with co-morbid PTSD and psychosis across five sites. Randomisation will be in the ratio 1:1 to the two groups and will be stratified by centre. It will be a pragmatic clinical and cost-effectiveness superiority trial.

An internal pilot study will ensure the integrity of trial recruitment; protocol safety and adherence; and outcome data [60]. The internal pilot will last 16 m following the start of recruitment and will include three checks for each of these categories, at 6, 12, and 16 m post recruitment start.

## Methods: participants, interventions, and outcomes

### Study setting {9}

There will be five recruiting sites across England (South London and Maudsley (SLaM); Greater Manchester Mental Health; Cumbria, Northumberland, Tyne and Wear; Oxford Health; Sussex Partnership), all of which are foundation NHS mental health trusts and have close links with Higher Education Institutions (HEIs) (King’s College London (KCL); University of Manchester; Newcastle University; Oxford University; University of Sussex). All secondary and tertiary care services within these trusts are potential sources of recruitment. Neighbouring trusts will be approached to act as Participant Identification Centres (PIC) for the recruiting sites if required.

The five NHS Trusts serve an ethnically diverse population and provide mental health care across a range of services in a variety of settings, ranging from adult Community Mental Health Teams, including Early Intervention for Psychosis services; inpatient wards and residential services; outpatient clinics and allied third sector services. Therapy will be delivered in community settings.

#### Covid-19 adaptations to the protocol

In response to the global pandemic, the research team has made adaptations to the protocol to enable the trial to continue in the event of social distancing restrictions being in place. Recruitment, assessment, and therapy delivery will be conducted remotely, using videoconferencing or telephone, where it is not possible to see participants in-person. Study documents such as Participant Information Sheets and trial leaflets will be sent either by e-mail or post. Wherever possible, participant choice for mode of attendance will be respected, unless it conflicts with NHS trust and University guidance at any given time.

### Eligibility criteria {10}

The target population will be adult mental health patients in secondary or tertiary care at the time of referral, presenting with current distressing psychotic and post-traumatic stress symptoms. They will meet diagnostic criteria for schizophrenia-spectrum diagnoses (SSD) and PTSD, the former determined by the research team following clinical notes review and consultation with care team, as appropriate, and the latter determined by a diagnostic assessment using a standardised measure.

Potential participants with psychosis who report a past index trauma, defined as event(s) experienced at least 1 m ago and still affecting them now (ascertained using the Mini-Trauma And Life Events (TALE) checklist (5 items depicting common traumas + 1 item in two parts (‘do any of the event(s) reported still affect you now and if so which one(s) currently affect you most’) [61]), will first be screened for the presence of at least one of the five re-experiencing items from the PTSD Checklist for DSM-5 (PCL-5 [33];, to ensure participants are presenting with PTSD-specific symptoms on which to anchor the trauma-focused therapy. Participants who satisfy the re-experiencing symptom criterion will then be administered the full-length TALE checklist [62], to determine trauma nature and timing, and elicit any other index traumatic event(s). They will then undergo a PTSD diagnostic interview based on the identified index trauma(s), according to the DSM-5 [35] criteria, to ensure presence and stability of PTSD symptom criteria.

**Fig. 1:**
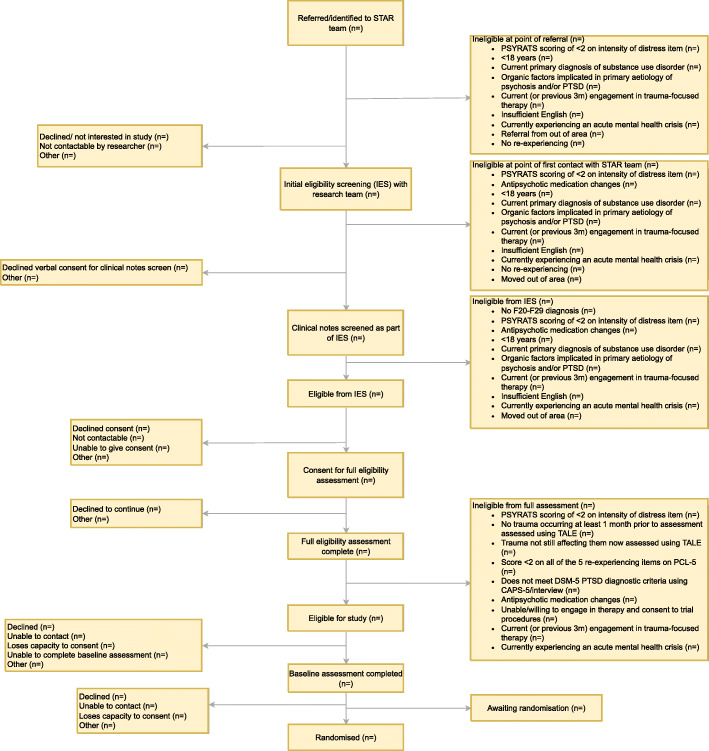
CONSORT diagram: Study of Trauma And Recovery (STAR) trial

People with SSD do not necessarily experience continuous psychotic symptoms, such as delusions and hallucinations, which typically are the targets of psychological therapies: their symptoms may have remitted, or they may present with cognitive or functional impairments only. We will therefore have the additional requirement that individuals report current distressing hallucinations and/or delusions (over the past month), as specified by previous CBTp trials [63, 64]. The full Consolidated Standards of Reporting Trials (CONSORT) diagram depicting the eligibility and assessment stages is presented in Fig. 1.

These specifications will ensure inclusion of people with at least moderately severe and stable post-traumatic stress and psychotic symptoms. Traumatic events can occur before or after psychosis onset, and co-morbid PTSD presentations are present in all stages of psychosis presentations. We will therefore not place any restrictions on type or timing of traumatic exposure, or participants’ age (apart from those applied to adult services).

#### Inclusion/exclusion criteria

##### Inclusion

Potential participants must meet the following criteria to be eligible:

(i) Presence of SSD (F20-29 diagnoses; International Statistical Classification of Diseases and Related Health Problems, 10th Edition; (ICD-10 [65];) from clinical notes review using the ICD-10 checklist [66], if necessary supplemented by information from the care team.
(ii) Scoring 2 or above (‘moderate’ intensity) on the intensity of distress item of the Delusions and/or Hallucinations Psychosis Symptom Rating Scales (PSYRATS [67]), adapted to include hallucinations in all modalities;
(iii) Reporting past trauma(s), occurring at least 1 m prior to assessment, including those related to psychosis or its treatment [68], assessed using the mini-TALE and TALE Checklists [62];
(iv) Reporting being currently affected by at least one past traumatic event, assessed using the mini-TALE and TALE Checklists [62];
(v) Scoring 2 or above (‘moderately’) on one of the five re-experiencing items from the PCL-5 [33]
(vi) Meeting DSM-5 [35] symptom criteria for PTSD diagnosis, assessed using the Clinician-Administered PTSD Scale for DSM-5 (CAPS-5 [69]), which includes the criteria of 1 m stability of symptoms and demonstrable link between the index trauma event(s) and presenting symptoms;
(vii) Both individuals on antipsychotic treatment, and those who decline to take medication, will be included, as long as no major medication changes have occurred in the previous 3 m (i.e. having started or stopped antipsychotic medication, or a switch to or from Clozapine);
(viii) Aged 18 and above;
(ix) Able and willing to engage in psychological therapy and consent to study procedures.

##### Exclusion

(i) Current, primary diagnosis of substance use disorder;
(ii) Organic factors implicated in the primary aetiology of psychosis and/or PTSD;
(iii) Current (or in previous 3 m) engagement in trauma-focused therapy (i.e. any therapy that focuses on reprocessing trauma memories; therapies such as CBTp would be operationalised as ‘trauma-informed’ rather than ‘trauma-focused’, since they may include past traumatic experiences in the developmental formulation or address the impact of events on appraisals and coping, but would not include memory work);
(iv) Insufficient English to provide informed consent or complete assessments without the help of an interpreter;
(v) Currently experiencing an acute mental health crisis.

### Who will take informed consent? {26a}

Potential participants will initially be contacted by a member of their clinical team in the participating or PIC NHS sites, who believes them to be suitable, inviting them to learn more about the study. They may also be contacted if they have indicated they are interested in research through their NHS Trust (e.g. ‘Consent for Contact’) and can also self-refer directly into the study. Once they have expressed their interest or agreed to be contacted, they will be approached by a Research Worker (RW) who will confirm eligibility criteria and obtain consent to participate in the research. Because of the sensitive nature of the questions for determining eligibility, informed consent will be obtained prior to eligibility assessments.

### Additional consent provisions for collection and use of participant data {26b}

All trial participants will be asked whether they would be willing to be contacted at a later stage for participating in further studies related to the trial or local research studies. This consent to contact will include the qualitative interview regarding the acceptability of the intervention, should they be randomised to the intervention group. It will be made clear that this is optional and that declining consent to be contacted for participation in additional studies will not prevent them from taking part in the trial.

Participants will also be asked whether they would be willing for their anonymised therapy recordings and quotes to be used for teaching, training, and other dissemination purposes.

## Interventions

### Explanation for the choice of comparators {6b}

The comparator will be TAU, which consists of multi-disciplinary psychologically informed care, delivered by mental health services.

Specifically, TAU will include standard psychiatric care consisting of medication and outpatient psychiatric appointments; psychologically informed case-management, including regular meetings with a care coordinator; access to a range of psychotherapies, which could include CBTp. Clinicians involved in participants’ treatment will receive a manual summarising current best practice and evidence-based treatment guidelines to promote standardisation of good quality TAU.

### Intervention description {11a}

#### Trauma-Focused Cognitive Behaviour Therapy for psychosis (TF-CBTp)

TF-CBTp is a manualised therapy integrating standard psychological therapy for PTSD and for psychosis [3, 8, 20, 25–27, 31]. It will be delivered over a period of 9 m. Approximately 26 weekly or bi-weekly individual 60–90-min sessions will be offered in the first 6 m. Bi-weekly rather than weekly sessions have a potential to be beneficial during the trauma reprocessing phase of the therapy [70, 71] but it will be left to participant choice. A further three sessions will be offered on a monthly basis during the next 3 m, to consolidate therapeutic gains. They will be done jointly with the care coordinator, if possible, to assist with generalisability of therapy effects. Therapists will liaise with clinical teams throughout the delivery of therapy to discuss progress, with the participant’s consent, and to share any potential risk to self or others.

**Fig. 2:**
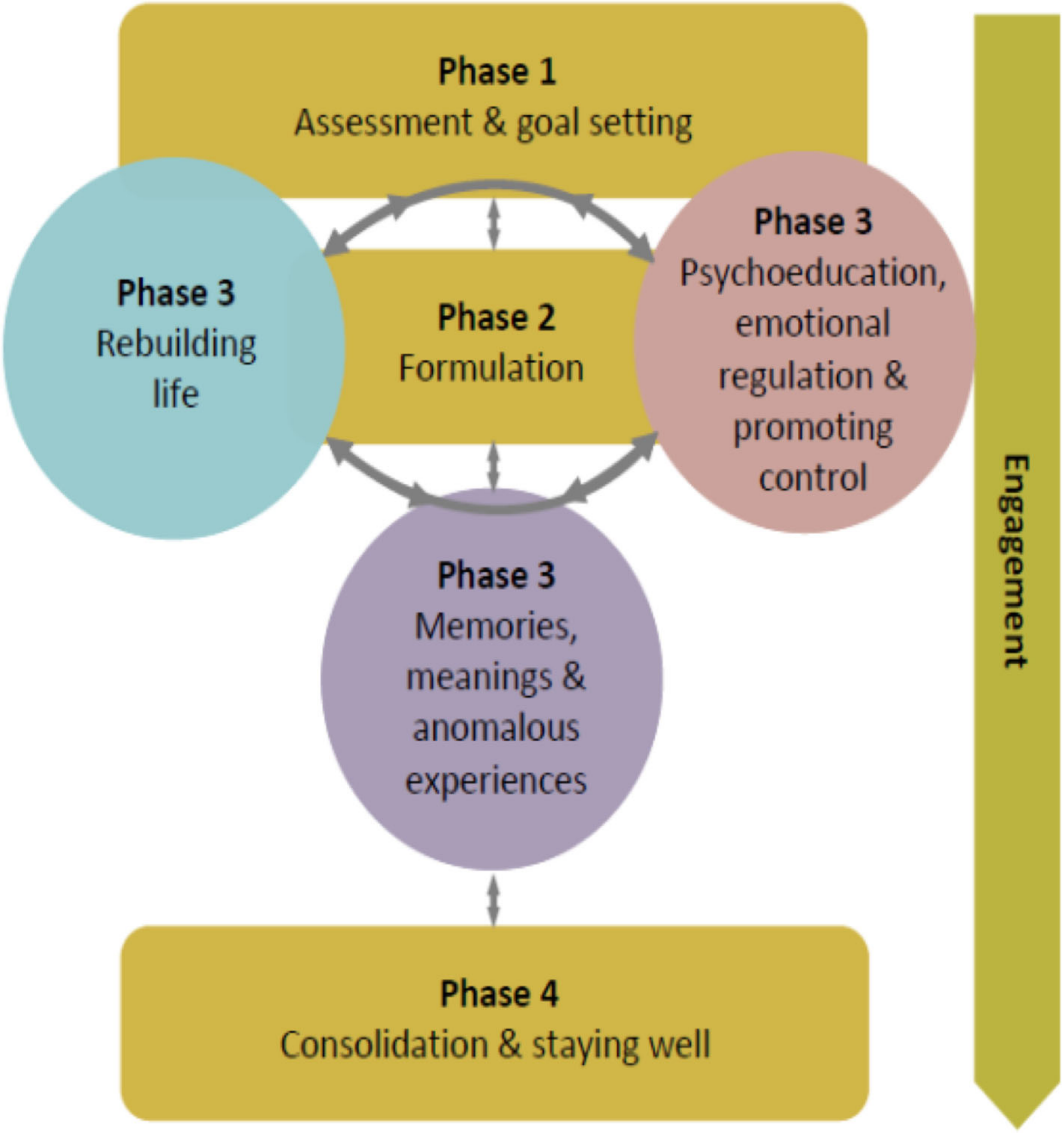
Trauma-focused cognitive behaviour therapy for psychosis (TF-CBTp) overview

TF-CBTp is formulation-based and individualised to tailor to the specific needs of the individual, depending on the type of PTSD presentation (for example, PTSD following a single traumatic event versus complex PTSD as a result of polyvictimisation) and type of psychotic symptom (for example, hallucinations or delusions). Therapy is conducted in a flexible style with an emphasis on engagement and building a good therapeutic relationship, which is key throughout the delivery of therapy. Trauma-focused work can be emotionally challenging, and throughout, a balance is struck between ensuring the person is able to manage distress and improve their coping if necessary, whilst not delaying or avoiding the trauma-focused interventions. Overall, TF-CBTp consists of four broad, flexible phases: (1) assessment, psychoeducation, and goal setting; (2) developing a shared understanding of current difficulties and maintenance cycles, i.e. formulation; (3) formulation-driven model-based interventions, consisting of (a) promoting control; (b) addressing trauma memories, anomalous experiences, and associated meanings; and (c) rebuilding your life, with cognitive, behavioural, and interpersonal techniques integrated as necessary into model-based interventions; and (4) consolidation and staying well (see Fig. 2). Phase 3 includes the memory reprocessing strategies, which are hypothesised to be necessary for the reduction of post-traumatic stress symptoms. The aim here is to reduce re-experiencing and/or associated psychotic symptoms through elaboration of the trauma memory and discrimination of triggers. The method of elaboration will be determined by the nature of the person’s memory intrusions as specified in the formulation but will include established reprocessing strategies such as imaginal reliving and imagery rescripting.

Given the time limited nature of the therapy and that the main target of therapy is post-traumatic stress symptoms, the aim is to adhere to PTSD model-based interventions unless adaptations for psychosis are necessary. In practice, therapy is formulation based and hence personalised and pragmatic in that it is adapted to the individual, with clinicians able to shift focus according to clinical need. Similarly, therapy speed and progression are tailored to the individual. Psychosis-focused interventions are embedded throughout to address psychosis-related experiences, appraisals, and behaviours as they arise.

### Criteria for discontinuing or modifying allocated interventions {11b}

There are no trial criteria for discontinuing or modifying allocated interventions at the individual participant level. It will be made clear to each participant that, should they find any aspect of the research distressing, and/or no longer wish to continue with either the research or the therapy, they will be able to withdraw from either or both without having to give a reason or this impacting on their usual clinical care in any way. Clinical teams will be responsible for the provision of TAU interventions, with no interference from the research team.

At the trial level, it is an important subsidiary goal of the trial to establish the safety of the intervention, and we will take all appropriate steps during the conduct of the trial for ensuring participant safety, in both arms of the trial. Concerns over safety of TF-CBTp, identified through adverse events (AEs) and serious adverse events (SAEs), therapy sessional ratings or qualitative interviews, would, in the first instance, lead to therapy protocol amendments, but could lead to study termination at any time. Our experience with this population and type of therapy suggests that the therapy proving unacceptable or too distressing to participants is a low risk. So far the evidence suggests the opposite, with van den Berg and colleagues [72] showing that fewer SAEs, symptom exacerbations, and revictimisation experiences were reported in the therapy groups, compared to the Waiting List group, suggesting that therapy decreases risk. However, we would see this unlikely eventuality as an important outcome of the study, as it would provide empirical evidence to inform future studies on what should be avoided in people with PTSD and psychosis, rather than relying solely on clinical intuition.

### Strategies to improve adherence to interventions {11c}

The intervention will be delivered by trained psychological therapists with experience of working with severe mental health problems, to ensure competence in engaging this complex population. They will receive training in delivering TF-CBTp by the study team (3 days training, with 2-day booster sessions in subsequent years). Therapists will travel to participants’ team base or home/residence if required, or conduct sessions remotely, to maximise retention in the therapy.

Three related aspects of therapy fidelity will be monitored and assessed: (i) participants’ adherence to the therapy, (ii) therapist adherence to the manual, (iii) therapist competence in delivering the therapy.

Participants’ adherence to the therapy will be assessed by recording the number of sessions offered and attended, including length of sessions attended. Completion of between-session tasks (i.e. therapy ‘homeworks’) will also be monitored.

Study-specific therapist adherence and competency scales will be developed to fit the therapy manual. Adherence will consist of a checklist of therapeutic procedures and therapy milestones extracted from the manual, to record content of sessions in terms of agenda targets, homework tasks, and change strategies used in all phases of the manual. Competence in applying these procedures will be assessed by adapting items from existing measures to assess skills and competences related to basic CBT, psychosis work, and PTSD trauma-focused work (PTSD-adapted version of the Cognitive Therapy Scale – Revised (CTS-R) [73], available at oxcadatresources.com).

To monitor therapy adherence, therapists will complete the adherence checklist following each session. These data will be extracted at regular time points throughout the trial to check therapy milestones are being met and ensure the therapy protocol is being followed. This ongoing monitoring will pick up on any adherence issues across sites or individual therapists and will inform training and supervision content.

Therapist competence in delivering the manual will be monitored through recordings of therapy sessions, with participants’ consent. Therapists will have weekly supervision from a senior clinical psychologist on each site, who will listen to and provide detailed feedback on a selection of therapy tapes using the competency scale to inform their feedback. All therapists and site supervisors will also meet remotely with the research team therapy leads for monthly group supervision. This will be done throughout the therapy delivery period to provide quality assurance and ensure action can be taken if required.

Therapy tapes from five participants on each site (total of 25 participants) will be randomly chosen to be rated by an expert clinician independent from the trial, to provide an objective verification of therapist fidelity, using the adherence and competency scales developed for the study. Six random tapes per participant, stratified by stage of therapy (with at least three tapes from Phase 3 where the formulation-driven model-based interventions occur) will be rated (total of 150 tapes).

### Relevant concomitant care permitted or prohibited during the trial {11d}

Participation will not alter clinical treatment decisions about medication, additional psychological and psychosocial interventions, or discharge to primary care or other care pathways, which remain the responsibility of the clinical team. The antipsychotic medication prescribed to participants in the study and psychological and psychosocial interventions provided will be recorded.

### Provisions for post-trial care {30}

There is no provision for post-trial care in the study, and participants will remain under the care of their usual mental health team or primary care services (if discharged from secondary care during their participation in the trial). The trial is covered by the Sponsor’s (King’s College London) indemnity insurance.

### Outcomes {12}

The primary outcome for the study is PTSD symptom severity at 9 m post-randomisation (end of therapy), assessed on the CAPS-5 [69] (past-month version), a semi-structured interview assessing the severity of symptoms delineated in DSM-5 [35]. It is currently the recommended clinical interview in PTSD research, including in psychosis populations [19]. The total symptom severity score will be the specific measurement variable.

Our secondary outcomes will consist of a range of clinical domains that are anticipated the therapy may impact on, namely percentage of participants achieving loss of PTSD diagnosis and demonstrating clinically significant change; self-reported PTSD and complex PTSD (Disturbances in Self-Organisation; DSO) symptoms; psychosis symptoms; emotional well-being, suicidal ideation and substance use; psychological recovery; and social functioning. Total or sub-scale scores for each questionnaire will be the measurement variable. See ‘Data collection and management’ section for a full list and description of measures.

We will collect further information on participants and their trauma experiences to characterise the sample, namely demographic variables (age; gender; ethnicity; migrant and asylum status; relationship status; education; living situation; working status); clinical variables (F20-F29 diagnosis); percentage of individuals reporting delusions and hallucinations (including multi-modal hallucinations); percentage who meet criteria for complex PTSD; lifetime substance use; age at start of psychotic symptoms and at first contact with mental health services; number of past psychiatric admissions; psychotropic medications); type of trauma experienced, timing, and multiple exposure, for all trauma experiences and the index event; and percentage of index events that meet Criterion A (exposure to actual or threatened death, serious injury, or sexual violence) on CAPS-5 [69].

In line with the recent CONSORT - Social and Psychological Interventions (CONSORT-SPI) [74] guidance, which recommends minimising the distinction between primary and secondary outcomes for psychological therapies trials, all outcomes will be reported at all assessment time points.

In relation to cost-effectiveness, we will collect data on service use and health-related quality of life at all assessment time points. In addition, service use will be assessed for the previous 3 months at the ‘keeping in touch’ phone calls between the end of therapy (9 m post-randomisation) and final follow-up (24 m post-randomisation), to maximise the accuracy of these data. See ‘Economic analysis’ section for further details.

**Fig. 3.**
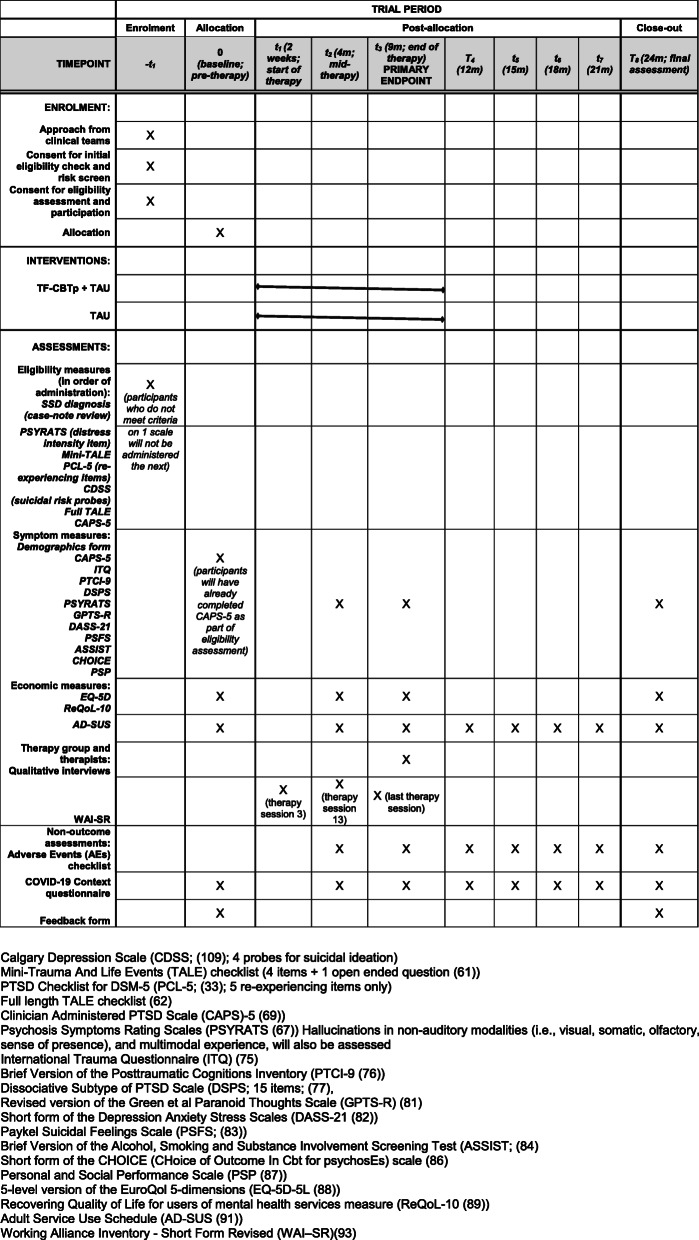
Schedule of enrolment, interventions, and assessments

Acceptability of the therapy, through qualitative interviews, will also be assessed as a secondary outcome in the therapy group, and in therapists delivering the therapy. This qualitative element will identify key aspects of acceptability and tolerability in receiving and implementing the therapy that could not be detected by quantitative measures alone. Therapeutic alliance will also be assessed in the intervention group only (Fig. 3).

### Participant timeline {13}

Research team members will conduct all eligibility and research assessments; all follow-up assessments will be conducted by research workers blind to study group. Therapy will last 9 m in the intervention group. Participants will remain enrolled in the study for 2 years in total.

Research assessments to assess PTSD and secondary outcomes, including cost-effectiveness outcomes, will take place at four time points (baseline, 4 m (mid-therapy), 9 m (end of therapy; primary endpoint), and 24 m (15 m post-therapy) post-randomisation). There will also be four ‘keeping in touch’ phone calls where service use will be assessed and contact details updated (at 12 m; 15 m; 18 m; and 21 m, post-randomisation). The 24 m post-randomisation assessment will enable us to determine whether therapy effects endure on both our primary and secondary outcomes. The 4 m assessment and the phone calls will help retention into the trial and reduce loss to follow-up, by avoiding having lengthy periods of time in the study without contact from the research team.

Qualitative interviews with participants in the intervention group will occur once participants have concluded or chosen to end therapy. Qualitative interviews with therapists will occur once they have completed therapy with a minimum of two participants.

### Sample size {14}

We will recruit 300 people for the study, 150 in each group, and 60 per site.

Sample size calculations accounted for clustering in therapy arm with intraclass correlation (ICC) = 0.01 with 10 therapists over the trial period, each with an average of 12 participants and variation in the cluster size of 12; no clustering in TAU group; 1:1 allocation; 0.05 significance level; baseline-endpoint correlation of 0.5 (range in previous data [19, 25]: 0.4–0.7) reducing the standard deviation (SD) to 0.866 from a standardised value of 1.

Allowing for 20% attrition to primary endpoint, 120 people per group in the analysis set has 91% power to detect an effect size of 0.4. As a sensitivity check, with 240 participants in the analysis set, if the ICC = 0.05 we would still retain 85% power. Alternatively, if the ICC = 0.01, a sample size of 240 participants has 80% power to detect an effect size of 0.33.

For the qualitative study, we will recruit 35 participants (7 per site) and between 5 and 10 therapists (1–2 per site, depending on number of therapists employed per site). At least five participants (one per site) who chose to end therapy prematurely will be included in the interviews.

### Recruitment {15}

Recruitment will occur in all services providing mental health care across the five participating NHS Trusts, to ensure as wide a range of ages and clinical presentations as possible. Participants will be identified through close liaison with clinical staff. After clinical staff have confirmed that a potential participant is suitable to be approached (i.e. has the potential to meet study criteria and no clinical contraindications), research workers will meet each potential participant to discuss the study, provide written information and time to consider it, respond to questions, and seek written informed consent.

We also intend to use recruitment databases or ‘consent for contact’ initiatives where available to maximise the pool of potential participants. Finally, we can be approached directly by service users interested in taking part and intend to place recruitment posters in the main clinical areas of specialist mental health teams, as well as promoting the trial through online platforms (Twitter, trial website) to facilitate this. In all such instances, we will contact the relevant clinical team and discuss suitability for participation.

A sub-sample of participants in the TF-CBTp group, who give their consent, will be consecutively recruited across all sites in the study to take part in a qualitative interview.

## Assignment of interventions: allocation

### Sequence generation {16a}

Randomisation will be in the ratio 1:1 to the two groups and will be stratified by centre. Randomisation (at the individual level) will be independent and concealed, using dynamically generated permuted blocks of random size.

### Concealment mechanism {16b}

The allocation sequence will be held independently of the research team and each participant’s allocation revealed via a secure web-based service hosted by King’s Clinical Trials Unit (KCTU). The allocation is dynamically generated and uses randomly varying blocks of sizes not known to the study team so allocation concealment is assured.

### Implementation {16c}

The research workers will enroll participants, and KCTU will assign participants to the two groups. The therapists will inform the participants to which group they have been randomised.

## Assignment of interventions: Blinding

### Who will be blinded {17a}

Clinicians, therapists, and participants will be unblinded, as is customary in psychological therapies trial. The research workers who conduct follow-up assessments will remain blinded to the allocation of participants until after the participant has completed their involvement in the trial. They will not be exposed to clinical notes or therapy records whilst they remain blinded. A system of web-based data entry will be used to ensure assessors do not have access to information in the database that might reveal allocation. Participants and clinical teams will be reminded prior to each assessment timepoint by the research team that they must not inform the research workers of their group allocation. The senior trial statistician will be blind throughout the study; the trial statistician will be unblinded at a group level after the first Data Management and Ethics Committee (DMEC) meeting. The Statistical Analysis Plan will be prepared before any unblinding of the trial statistician, and only amended by the senior trial statistician.

### Procedure for unblinding if needed {17b}

There are no pre-determined situations in which blinded research workers should become unblinded. Breaks in blindness will be monitored and recorded and where operationally feasible assessments will be allocated to another (blinded) research worker. Where blind breaks occur during an assessment, any interview-based measures that have been recorded will be scored by another (blinded) research worker, removing the unblinding information.

## Data collection and management

### Plans for assessment and collection of outcomes {18a}

#### Procedure for assessments

Trained team members will conduct all assessments at the four time points and all ‘keeping in touch’ phone calls, supervised by experienced research clinical psychologists. All follow-up assessments and phone calls will be conducted by research workers blind to study group.

Assessments will be conducted at locations convenient for the participant (at either NHS, University or residential locations, or remotely). Participants will receive a reimbursement of £20 for completing each research assessment and £10 for the qualitative interview, plus travel expenses. Participants will be offered choices regarding length of assessments, including the option of breaks and multiple testing sessions.

#### Data quality

Assessors will be trained to competence on the interview outcome measures (CAPS-5 and PSYRATs scales) [67, 69] prior to starting any assessments, i.e. they will need to have reached > 80% agreement with ratings made by the Trial Coordinators on training videos during the initial training stage. Agreement will be based on symptom presence ratings for the CAPS 5 (i.e. scores of 2 or above for each item of criteria B-E) and individual item scores for the PSYRATS. Once started, assessments will be recorded, with participants’ consent, to conduct further inter-rater reliability on the interview measures. Each research worker’s assessments will be double-rated by the Trial Coordinator until > 80% agreement has been reached. These procedures will be repeated every 4–6 months to minimise rater drift. Inter-rater reliability for our primary outcome will be reported from the double ratings made throughout the lifetime of the trial for a minimum of 60 ratings (excluding those used for rating agreement during the initial competency training stage prior to obtaining > 80% agreement).

#### Measures

All measures were selected or designed with the participant population in mind and are considered suitable for people with psychosis to complete. A range of potential measures for each domain of assessment was presented to our experts-by-experience advisory group who made the final selection, based on consideration of content acceptability and burden. An extra questionnaire will be administered to capture contextual information relating to the Covid-19 pandemic such as the degree of restrictions at the time of assessment, level of lockdown in the previous month, and how much Covid-19 has impacted on the person.

##### Primary outcome

PTSD symptom severity in the past month will be assessed on the CAPS-5 [69]. The CAPS-5 is a semi-structured interview assessing the severity of symptoms delineated in DSM-5 [35].

The CAPS-5 consists of seven criteria (Criteria A to G). Scores are anchored to an index event, which will be elicited using the mini-TALE and TALE checklists [61, 62]. The index event could be a single trauma experience or multiple incidents. In this study, meeting Criterion A (i.e. only events including objective actual or threatened death, serious injury, or sexual violence) will not be a requirement, as we will include events related to psychosis and its consequences (e.g. hearing a threatening voice, involuntary admission or forced restraint), emotional and physical neglect, discrimination, and attachment disrupting experiences, as possible index events [68]. PTSD diagnostic status will be determined by an algorithm of minimum scores on specific items from Criteria B to E, and meeting Criteria F and G, according to DSM-5 diagnostic rules.

Once the index event has been ascertained, the severity of symptoms is scored on a 5-point scale (‘absent’ to ‘extreme’) on four criteria: (i) Criterion B: Re-experiencing symptoms; (ii) Criterion C: Avoidance symptoms; (iii) Criterion D: Cognitions and mood symptoms; (iv) Criterion E: Arousal and reactivity symptoms. Criteria F and G are scored dichotomously (Yes/No) on whether the duration of the experience is more than 1 m, and has caused subjective distress and impairment in functioning, respectively. The total symptom severity score (total of 20 item scores on Criteria B to E) will be the primary outcome.

##### Secondary outcomes

All measures listed below consist of standardised questionnaires and semi-structured interviews, with demonstrated reliability and validity. Short forms have been included when available, to minimise participant burden. The timescale of assessment will be the past month for all symptom measures, consistent with our primary outcome (apart from substance use, which will be 3 months as the timescale cannot be amended, and the economic measures). All have been used in previous trials with psychosis populations and were endorsed by our experts-by-experience advisory groups.

#### Symptoms

##### PTSD

PTSD includes the following: (i) percentage of people who achieve a loss of their PTSD diagnosis, as determined by the CAPS-5 diagnostic status algorithm; (ii) percentage of people who show a clinically significant improvement and a reliable change in CAPS-5 scores; (iii) CAPS-5 individual symptom clusters (severity scores for the individual Criteria B to E); (iv) self-reported PTSD symptoms and their associated appraisals and responses will be assessed on standardised, commonly used questionnaires: International Trauma Questionnaire (ITQ [75]); PTSD and DSO dimensional scales (6 items each); Brief Version of the Posttraumatic Cognitions Inventory (PTCI-9 [76]; 9 items), which measures cognitive appraisals of the trauma and its aftermath; Dissociative Subtype of PTSD Scale (DSPS; 15 items [77];), which assesses lifetime occurrence and current frequency and intensity of dissociative symptoms (consisting of three factors of dissociation, namely psychogenic amnesia, derealisation/depersonalisation, and loss of awareness).

##### Psychosis

(i) The Psychosis Symptoms Rating Scales (PSYRATS [67]) is a clinician-administered semi-structured interview and will be used to assess the multidimensional aspects of delusions and auditory hallucinations (such as distress, preoccupation, and conviction; 11 items for voices, and six items for delusions). The PSYRATS is well suited to assess outcome in psychological therapies [78] and has been used in major RCTs [63, 64]. PSYRATS items will also be administered for hallucinations in other modalities (i.e. non-verbal auditory; visual; somatic; sexual sensations; olfactory; gustatory; sense of presence [79]), and additional items will be included at the baseline assessment to assess multi-modality (i.e. whether different types of hallucinations are experienced simultaneously or serially and are related or unrelated) [80].

Each PSYRATS item is rated by the interviewer on a 5-point nominal scale (0–4). An additional, continuous self-report rating scale will be added to each item, as there is evidence that the nominal scale is not sensitive to change for some of the items (e.g. delusional conviction is rated as a 3 for conviction ratings of 50–99%, and as 4 for 100%; therefore, a 50% change in delusional conviction only incurs a 1-point difference). The rating scale will be presented in the form of a thermometer to facilitate self-report. Both ratings (total scores for each PSYRATS scale) will be reported as secondary outcomes.

(ii) Self-reported paranoia (the commonest form of delusions) will be assessed using the Revised Green et al Paranoid Thoughts Scale (GPTS-R [81];; 18 items).

##### Emotional well-being

(i) Mood will be assessed using the short form of the Depression Anxiety Stress Scales (DASS-21 [82]), which includes seven items for each of the three domains assessed. (ii) Suicidal ideation will be assessed using the Paykel Suicidal Feelings Scale (PSFS [83]; 5 items). (iii) Substance use will be assessed by the Brief Version of the Alcohol, Smoking and Substance Involvement Screening Test (ASSIST [84, 85];) developed by the World Health Organization (WHO). It comprises 10 items pertaining to lifetime (Part 1) and recent (Part 2) use of substances.

##### Psychological recovery

Psychological recovery will be assessed using the Short Version (11 items + 1 personal goal) of the CHOICE (CHoice of Outcome In Cbt for psychosEs) scale [86]. CHOICE was developed by our group in collaboration with experts-by-experience, reflecting themes they considered important psychological therapy outcomes.

##### Social and occupational functioning

This will be assessed using the Personal and Social Performance Scale (PSP [87]). It is a 100-point single-item rating scale based on the assessment of functioning in four areas (socially useful activities, personal and social relationships, self-care, and disturbing and aggressive behaviour). For an impairment to be rated, it must relate to psychological problems rather than lack of opportunity.

##### COVID-19 context questionnaire

Contextual information will be captured using a 7-item scale assessing mode of assessment and current level of pandemic restrictions; personal lockdown circumstances; and impact of the pandemic on day-to-day life, well-being, and PTSD and psychosis symptoms.

#### Economic measures

##### Health-related quality of life

Health-related quality of life will be measured using the 5-level version of the EuroQol 5-dimensions (EQ-5D-5L [88]), introduced by the EuroQol Group as an alternative to the standard EQ-5D-3 L, to provide greater sensitivity and to reduce ceiling effects. The EQ-5D-5L descriptive system comprises five dimensions (mobility, self-care, usual activities, pain/discomfort, and anxiety/depression) each with five levels (no problems, slight problems, moderate problems, severe problems, and extreme problems). Participants are asked to respond on the basis of which answer best describes their health ‘today’. The score for each dimension can be combined into a 5-digit number that describes the person’s health state.

##### Health-related quality of life for users of mental health services

We will additionally include the Recovering Quality of Life (ReQoL-10 items [89]), which may be more sensitive to change than the EQ-5D in populations with severe mental health problems [90]. Respondents are required to answer on a 5-point scale assessing their thoughts, feelings, activities, and physical health over the last week.

##### Service use

Service use for costing purposes will be measured in interview using a modified version of the Adult Service Use Schedule (AD-SUS), designed, and successfully applied in psychosis populations [91]. The STAR AD-SUS will measure use of all-cause health and social services appropriate for the NICE preferred NHS/Personal Social Services perspective [92].

#### Therapy group only

##### Acceptability

A qualitative interview will be designed to explore participant acceptability and satisfaction with the therapy. Close attention will be paid to any emotional distress resulting from memory reprocessing procedures, in particular potential impact on psychotic symptoms, and whether this was considered unacceptable or unnecessary. The views of those who chose to end therapy early will be gathered at point of ending, using additional questions about their reasons for doing so and to identify barriers and potential solutions to engagement in therapy. Therapists will be interviewed once they have completed therapy with a minimum of two participants to obtain feedback about acceptability, and any potential difficulties in delivery.

Experts by experience researchers with lived experience of psychosis will conduct the participant interviews, with appropriate supervision and support, and therapists will be interviewed by research workers. It is anticipated that the final patient sample will be representative and include variance on key variables (e.g. therapy engagement, age, gender, ethnicity, clinical presentation). All interview data will be recorded, with participants’ permission, and transcribed verbatim for analysis.

##### Therapeutic alliance

We will assess the therapeutic alliance between therapists and participants in the therapy group to monitor the potential impact of different modes of therapy delivery, implemented as a result of the Covid-19 pandemic, on the therapeutic relationships. The therapists and client versions of the Working Alliance Inventory – Short Form Revised (WAI-SR) [93] will be used. Three key aspects of alliance are assessed: agreement on therapy tasks, agreement on therapy goals, and the development of an affective bond [93]. Both the self-report participant and therapist versions will be completed at three time points (sessions 3, 13, and last session). The therapists will be blind to participant ratings at all time points.

### Plans to promote participant retention and complete follow-up {18b}

A number of strategies are planned to maximise participant retention into the trial and ensure completeness of outcomes. A ± 1 m window will be allowed for completion of assessments at each time point. Assessment measures will be clearly prioritised so that the most important will be collected first to avoid missing data. The CAPS-5 [69] (primary outcome) will always be administered first, followed by the PSYRATS [67] and the health economy measures.

Participants who choose to end the therapy early or deviate from the allocation protocol (e.g. someone receiving trauma-focused therapy in the TAU group) will still be invited to complete all follow-up assessments. Participants will be remunerated for their time and travel, which secures good concordance with trial procedures, even in those who end therapy early [94]. There will be flexibility around the location of assessments, including remote assessments or home visits for participants who are unable to travel. Anyone who moves within the UK will be followed up.

A mid-therapy assessment is included to minimise attrition from the trial, as 9 m is a long period without contact with the research team for this population. This is particularly the case for the control group and those who end therapy early, and the extra assessment stage will provide data that can be used in the linear mixed model for the intention-to-treat analysis for those who drop out of the study at the primary endpoint. This adds some validation to a missing-at-random assumption for outcome missingness. The four ‘keeping in touch’ phone calls will also help to retain participants in the trial until the final assessment 24 m post-randomisation.

### Data management {19}

All data are anonymised at source. No patient identifiable information is recorded on the research assessment records, and the computerised database is held centrally and managed by the King’s Clinical Trials Unit (KCTU). A web-based electronic data capture (EDC) system will be designed, using the InferMed Macro 4 system. The EDC will be created in collaboration with the trial analyst/s and the Trial Coordinator and maintained by the KCTU for the duration of the project. It will be hosted on a dedicated server within KCL.

Source data will be entered by the research workers at each site by authorised staff onto the EDC. A full audit trial of data entry and any subsequent changes to entered data will be automatically date and time stamped, alongside information about the user making the entry/changes within the system. Database access will be strictly restricted through user-specific passwords to the authorised research team members. The data management plan is in the study site file and can be provided on request.

Data quality will be ensured by close monitoring and routine auditing for accuracy throughout the data collection period. To ensure the accuracy of the data entered into the database, the main outcome measure entry will be checked for 20% of participants by comparing the paper record with that on the database. An error rate of no more than 5% is acceptable. If the error rate is higher than 5%, the percentage of participants checked will increase to a minimum 50%. No data will be amended independently of the study site responsible for entering the data. Prior to analysis of the 9 m and 24 m outcomes, there will be a central process of data cleaning and checking to verify that all data are complete and correct. At this point, data can be formally locked for analysis of that timepoint.

Pseudonymised recordings of the qualitative interviews will be transcribed by a KCL-approved transcription service. All recordings will be transferred and stored securely, and the transcription service will follow GDPR regulations (2018).

### Confidentiality {27}

#### Clinical confidentiality

Issues relating to confidentiality will be addressed at the eligibility stage and potential participants will be advised of the limits of confidentiality. It is also possible that disclosure of criminal or other acts potentially requiring action will occur during assessment and therapy sessions. The research team will be trained in both local and national policies for dealing with such disclosures and will follow our Standard Operational Procedures for managing risk disclosures. Therapists will address confidentiality issues again with participants allocated to the TF-CBTp group at the start of therapy, and at any appropriate subsequent points during the therapy.

#### Data confidentiality

Research data will be confidential unless a participant discloses information that indicates that they or another person are at risk of harm. If harm is disclosed, the research worker or trial therapist would be required to share this information with the participant’s care team and documented in the NHS trust’s electronic patient record system.

All research data will be pseudonymised. A hard copy of a record sheet linking Participant Identifiable Data (PID) (participant identity, contact details, trial identification number) for all participants will be kept separate from the research data at each site. The participant record sheet, and details necessary for communication with clinical teams, will be placed securely in locked filling cabinets separate from research datasheets. During the COVID-19 pandemic, staff may have to work remotely outside of Trust premises; an electronic version of the record sheet linking PID will be created and saved on secure Trust drives accessible online.

All data will be kept secure at all times and maintained in accordance with General Data Protection Regulation (GDPR, 2018) requirements and archived according to clinical trial Good Clinical Practice regulations. Participant consent forms will be retained, kept confidential, and stored securely. All identifiable data will be destroyed following a period of 10 years (as determined by relevant information governance policies) after the completion of the trial.

No participant identifiable information is recorded on the research assessment records and the computerised database is held centrally and managed by the KCTU. Data from the assessments are entered into this central record by research workers using a secure network connection.

Therapy files will be kept in a secure office and are not accessible to the staff collecting the research outcome data.

#### Recordings

Encrypted recording equipment (such as encrypted smart phone, laptop, or equivalent devices) will be used to record assessments (with participant consent) to check fidelity to assessment protocols and allow for multiple ratings of assessments to ensure inter-rater reliability. The therapy sessions will also be recorded (with participant consent) for monitoring the fidelity of the intervention delivery. These files named with a unique participant identifier will be transferred to secure central storage as soon as possible and stored as computer files on secure NHS/ University servers. Recordings of the therapy will be accessible to the participant’s therapist, the supervisor, and a random selection to the independent fidelity rater. With additional, optional participant consent, anonymised therapy excerpts will be used for training and dissemination purposes.

The study will adhere to the joint guidance on secure recording issued by KCL and the NHS Trusts. When not in use, encrypted devices will be stored in a locked cabinet within a locked office. Each device will be password protected. In the event of the device being lost or stolen, this will be reported as a data incident to the Information Management and Compliance Team at KCL and the Information Governance Team at the relevant NHS Trust. Any sensitive data on a lost/stolen device will be remotely erased.

Pseudonymised audio recordings of the qualitative interviews will be transcribed by a KCL-approved transcription service. All recordings will be transferred and stored securely, and the transcription service will follow GDPR regulations (2018).

### Plans for collection, laboratory evaluation, and storage of biological specimens for genetic or molecular analysis in this trial/future use {33}

N/A

## Statistical methods

### Statistical methods for primary and secondary outcomes {20a}

#### Primary and secondary outcomes

We will report data in line with the CONSORT-SPI [74] statement showing attrition rates and loss to follow-up. All analyses will be carried out using the intention to treat principle, incorporating data from all participants including those who do not complete therapy. Every effort will be made to follow up all participants in both arms for research assessments.

Analyses will be conducted in Stata version 15 or later. Descriptive statistics within each randomised group will be presented for baseline values. These will include counts and percentages for binary and categorical variables, and means and standard deviations, or medians with lower and upper quartiles, for continuous variables, along with minimum and maximum values and counts of missing values. There will be no tests of statistical significance or confidence intervals for differences between randomised groups on any baseline variable.

Treatment effects on primary and secondary outcomes will be estimated using linear mixed models. Fixed effects will be centre, baseline assessment for the outcome under investigation, group, time (categorical), and time × group interactions. Participant and therapist will be included as random intercepts. Marginal treatment effects will be estimated and reported for each time point as adjusted mean differences in scores between the groups with 95% confidence intervals and 2-sided *p*-values. For binary secondary outcomes, the same approach will be followed using logistic mixed models and effects will be reported as conditional odds ratios.

The analysis will be conducted in two separate stages. The primary analysis will estimate treatment effects at the primary endpoint of 9 m, using all outcome data from 4 and 9 months. Analysis will take place after the last participant has completed their 9 m assessment and will report the estimated effects at 4 and 9 months. The results will not be shared with the blinded assessors, nor outside of the STAR team, until all 24-m assessments have been completed, to avoid any biases on data collection.

The secondary analysis will estimate treatment effects at 24 m, using all outcomes from 4, 9, and 24 months. Analysis will take place after the last participant has completed their 24-m assessment, and will report the estimated effect at 24 m.

Cohen’s *D* [21] effect sizes will be calculated as the adjusted mean difference of the outcome divided by the pooled sample standard deviation of the outcome at baseline. These will be displayed in a forest plot showing the therapy effects on the primary and the secondary outcomes at 9 and 24 months post-randomisation.

Missing data on individual measures will be pro-rated if more than 80–90% (depending on questionnaire) of the items are completed; otherwise, the measure will be considered as missing. We will check for differential predictors of missing outcomes by comparing responders to non-responders on key baseline variables. Any significant predictors will be included in the analysis models. This accounts for missing outcome data under a missing at random assumption, conditional on the covariates included in the model. As a sensitivity analysis, we will assess whether therapy adherence is associated with missing data and, if it is associated, use inverse probability weights or multiple imputation to compare results.

#### Economic analysis

A within-trial cost-effectiveness analysis will be carried out, taking the NHS/personal social services perspective preferred by NICE [92]. Service use will be measured in interview using the AD-SUS [91], at baseline (covering the previous 3 months), at the 4 m, 9 m, and 24 m follow-up points, and at the four 3-monthly phone calls (covering the period since previous interview/phone call, thus ensuring coverage of the full 24-m period). Service use will be costed using nationally applicable unit costs (e.g. NHS Reference Costs for hospital contacts; Personal Social Services Research Unit (PSSRU) Unit Costs of Health and Social Care for community-based services; and the British National Formulary for medication). The TF-CBTp intervention will be directly costed taking a standard micro-costing approach [95]. Data on therapist time will be collected from clinical records (number and duration of face-to-face contacts) and unit costs will be based on the mid-point of the therapists’ salary, including all employer costs (National Insurance and superannuation) and appropriate overheads (capital, managerial, administrative, etc.). The cost of supervision will be included and indirect time (for, e.g. training, administration, meetings with other professionals) will be estimated using a questionnaire completed by each therapist on the time they spend on various direct and indirect patient-related activities.

The primary economic evaluation will be a cost-utility analysis carried out at the 9-m endpoint (end of therapy), in line with the clinical analyses, with outcomes expressed in terms of QALYs, calculated from the EQ-5D-5L [88], using the area under the curve approach [96]. Given evidence to suggest the EQ-5D may not be particularly sensitive in psychosis populations, the new ReQoL measure [89] and the primary clinical outcome measure (CAPS-5 [69]) will be included in secondary economic analyses. All three economic evaluations will be repeated at the 24-m follow-up to explore the longer-term impact of TF-CBTp compared to TAU.

Costs and QALYs will be compared at the 9-m and 24-m follow-up points and presented as mean values by trial arm with standard deviations. Mean differences in costs and 95% confidence intervals will be obtained by non-parametric bootstrap regressions to account for the non-normal distribution commonly found in economic data [97]. Cost-effectiveness will be assessed using the net benefit approach and following standard approaches [98]. A joint distribution of incremental mean costs and effects for the two groups will be generated using bootstrapping to explore the probability that TF-CBTp is the optimal choice compared to TAU, subject to a range of possible maximum values (ceiling ratio) that a decision-maker might be willing to pay for unit improvements in outcomes. Cost-effectiveness acceptability curves will be presented by plotting these probabilities for a range of possible values of the ceiling ratio [99]. These curves are a recommended decision-making approach to dealing with the uncertainty that exists around the estimates of expected costs and expected effects associated with the interventions under investigation and uncertainty regarding the maximum cost-effectiveness ratio that a decision-maker would consider acceptable. To provide more relevant treatment-effect estimates, all economic analyses will include adjustment for the variable(s) of interest and baseline covariates [100], which will be pre-specified and in line with the clinical analyses. The primary analysis will be a complete case analysis with the nature and impact of missing data, in particular those lost to follow-up, explored in sensitivity analyses.

#### Qualitative interviews

All interview data will be analysed using thematic analysis [101], which results in a rich and accessible account of qualitative data. This process involves systematically and iteratively coding information from interviews under main headings and subcategories, and using previous literature to support the validity of categories. Member checking strategies [102] will be employed for this stage of the analysis with participants, members of the research team, and expert-by-experience consultants, to maximise the transparency and trustworthiness of the data. Data management and analysis will be supported by NVivo software. Analysis will occur in parallel with data generation and will continue until thematic saturation is achieved (the point at which no new categories emerge). All trial documentation and data will be retained for a minimum of 10 years, as stated in Clinical Trials Regulations.

### Interim analyses {21b}

No interim analyses are planned.

### Methods for additional analyses (e.g. subgroup analyses) {20b}

Any putative subgroups, including centre, will be assessed by including an interaction term between randomisation and the subgroup variable, as well as a main effect for the subgroup if not already in the model. All additional analyses will be clearly specified in the Statistical Analysis Plan and reviewed by the DMEC.

### Methods in analysis to handle protocol non-adherence and any statistical methods to handle missing data {20c}

The random effect structure of the main analyses will account for repeated measures and clustering due to the partial nested design. All models will be estimated using maximum likelihood estimation, which allows for missing outcome data under the Missing At Random assumption; we may also use inverse probability weighting to adjust for non-adherence to allocated treatment and other intermediate outcomes as predictors of future loss to follow-up. A dose-response model will be considered to estimate a linear effect of amount of therapy, with randomisation as an instrumental variable for the number of TF-CBT sessions attended. Complier-average causal effects will be estimated using instrumental variable methods or finite mixture models, using pre-specified definitions of compliance/fidelity in the TF-CBT arm.

### Plans to give access to the full protocol, participant-level data, and statistical code {31c}

The investigators will permit trial-related monitoring, audits, and Research Ethics Committee (REC) review by providing the Sponsors, the DMEC, and REC direct access to source data and other documents as required.

Anonymised datasets generated during and/or analysed during the current study will be available, and the corresponding statistical code, upon request post publication of the trial results from the Principal Investigator (PI), following review of appropriateness of request by the trial team.

## Oversight and monitoring

### Composition of the coordinating centre and trial steering committee {5d}

The trial has been carefully designed to ensure compliance with Good Clinical Practice and scientific integrity. The research programme development, design, and implementation will be managed by the PI and the co-applicants, in consultation with experts-by-experience consultants and other expert collaborators from within and outside of the PI’s institution. The trial will comply fully with KCTU Standard Operating Procedures. Dedicated Trial Coordinators will assist in the day-to day management of the project reporting to the PI. A Trial Management Group will meet monthly; its membership will include the investigators, the Trial Coordinators, and site leads. It will be chaired by the PI and will manage the day-to-day running of the study and ensure good communication between trial sites, receiving monthly reports from each site on recruitment, therapy completion, adverse events, reviewing progress against milestones, and finding solutions to problems as they arise. It will oversee the preparation of reports to the Trial Steering Committee (TSC) and DMEC.

The TSC will oversee the study on behalf of the trial Sponsor and Funder and ensure that the study is conducted within appropriate NHS and professional ethical guidelines. It will provide advice on all appropriate aspects of the project; will oversee progress of the trial, adherence to the protocol, participant safety, and the consideration of new information of relevance to the research question; will ensure the rights, safety, and well-being of the participants are given the most important considerations and should prevail over the interests of science and society; will ensure appropriate ethical and other approvals are obtained in line with the project plan; will agree proposals for substantial protocol amendments and provide advice to the sponsor and funder regarding approvals of such amendments. It will comprise six independent members: a chairperson, a clinician, health economist, statistician, and two experts by experience. The PI, Trial Coordinators, and Therapy Lead will join the meeting as observers.

### Composition of the data monitoring committee, its role, and reporting structure {21a}

The DMEC will monitor the following: (1) recruitment of study participants; (2) ethical issues of consent; (3) quality of data (including missing data and unblindings); (4) the incidence of serious adverse events (SAEs); (5) any other factors that might compromise the progress and satisfactory completion of the trial. The Chair will be responsible for confirming judgements on the likelihood of any SAE’s relatedness to trial procedures as well as the SAE’s intensity and unexpectedness. The DMEC will make recommendations to the TSC on whether there are any ethical or safety reasons why the trial should not continue, with the safety, rights, and well-being of participants being paramount. It will consider the need for any interim analyses, including potential requests from the Funder, and will advise the TSC regarding the release of data and/or information. The DMEC will consist of three independent members: a chairperson, a clinical academic, and a statistician. The PI, trial coordinators, and statisticians will attend parts of the DMEC meeting to provide reports but will not be members or be present when unblinded data is discussed. The DMEC charter is in the study site file and can be provided on request.

## Patient and public involvement (PPI) strategy

One of the co-applicants (EL) is an expert-by-experience researcher and will be involved at all stages of the research, from the application through research delivery to dissemination. She will be part of the Trial Management Group and will lead on the qualitative aspects of the study. In addition, we will have local PPI reference groups who will be asked to contribute to the delivery of the trial in a range of ways, e.g. consultation and piloting on assessment protocols; reviewing materials; supporting training of staff; supporting recruitment to the trial; conducting qualitative interviews with participants in the intervention group; and assisting with dissemination. Experts by experience researchers conducting the interviews will be provided with supervision from EL and site leads.

### Adverse event reporting and harms {22}

Best practice, professional guideline, and local NHS policies for monitoring mental state and risk will be followed throughout the participants’ involvement in the trial and will be facilitated by close liaison with clinical teams. The safety of the intervention will be monitored closely during therapy sessions and through regular contact with the participant’s clinical team or GP.

The occurrence of adverse events (AEs) will be monitored actively and systematically and recorded by research workers and therapists, following guidance from the CONSORT-SPI [74] with the extension for non-pharmacologic treatment, and the extension for reporting of harms. Reasons for withdrawal from the study will also be recorded. Medical Research Council Guidelines for Good Clinical Practice in Clinical Trials will also be followed to ensure good governance of the trial for integrity and participants’ safety and well-being.

To ensure active surveillance of harms, at each assessment point, research workers will actively check for the occurrence of specific AEs using a structured checklist completed with the participant. Clinical notes will additionally be checked by unblinded members of the research team for any further undisclosed record of AEs. This extra procedure is to ensure completeness of records and to address the possibility of an increased likelihood of disclosure of AEs in the TF-CBTp condition, as a result of greater frequency of contact and the therapeutic relationship.

Good Clinical Practice guidance for non-CTIMPs (Clinical Trial of an Investigational Medicinal Product) studies will be followed to make decisions regarding seriousness (i.e. AEs vs SAEs), relatedness to the trial (i.e. Related Events (REs) and serious related events (SREs), and unexpected serious related events (USREs).

AEs are defined by the Health Research Authority as any untoward medical occurrence, unintended disease or injury, or untoward clinical signs in participants, whether or not related to the treatment, which require additional support or input from health professionals. In addition, since an important subsidiary goal of the trial is to establish the safety of the intervention, issues specific to psychological therapies [103, 104], and specific concerns clinicians have about trauma-focused therapy with psychosis individuals, will also be monitored, namely clinically significant increases in mental health problems and/or risky or problematic behaviours; harm to self/others, including suicide attempts; harm from others; emergency room visits; or crises. Clinically significant increases will be operationalised as an unresolved exacerbation requiring increased involvement from the care team, e.g. requiring a change in treatment plan. Distress or complaints associated with therapy, completion of assessment measures, or any other trial procedure would also constitute AEs.

The causes for the AEs will also be recorded and monitored. For each AE, the following potential reasons will be identified: victimisation (aggressive behaviour, sexual abuse/assault, physical abuse/assault, emotional abuse/psychological maltreatment, exploitation, and other victimisation); mental health/psychological problems (excessive use of substances, general distress, psychotic symptoms, PTSD symptoms, suicidal ideation, and other psychological); trial procedures (group allocation, assessments, or therapy); physical health, including COVID-19; accidents or natural disasters; and other.

AEs will be assessed initially at three levels of severity, namely mild, moderate, and severe, which reflect the severity or impact of the event on the person at the time, and is distinct from seriousness. Seriousness relates to the outcome of the event and is the criteria for defining regulatory reporting obligations: SAEs are defined as death and life-threatening events (Category A), incidents which acutely jeopardise the health or psychological well-being of the individual, resulting in immediate hospital admission and/or persistent or significant disability or incapacity (category B), or resulting in injury requiring immediate medical attention (category C). However, in this study, any AE rated as ‘severe’ will automatically be classified as an SAE.

AEs will be categorised for severity and seriousness by the site leads/Trial Coordinators. Relatedness to the trial will be judged based on whether the event resulted from administration of any of the research or therapy procedures, according to a temporal relationship (i.e. SREs). Unexpectedness of an event to the intervention will be judged based on whether the event is unexpected or unexplained given the participant’s clinical course, previous conditions and history, and concomitant treatments (i.e. USREs).

Events identified as SAEs will be further reviewed by the PI initially and additionally by the chair of the DMEC. Only SAEs that have been judged by the PI and the chair of the DMEC to be USREs will be reported to the REC. The DMEC will be responsible for investigating further, if there are any concerns about unexpectedly high rates of SAEs, SREs, or USREs, which may include being unblinded as to trial condition or seeking further data on adverse events, and will advise the TSC on any ethical or safety reasons why the trial should be prematurely ended. The Funder will immediately be notified on receipt of any information that raises material concerns about safety or efficacy, and of any recommendations from the DMEC to end the trial.

### Frequency and plans for auditing trial conduct {23}

It is anticipated that the DMEC and TSC will be convened on a 6-monthly basis, but either the research team or the DMEC/TSC will have the opportunity to request an increased frequency of meetings, should it be indicated. The DMEC will receive open reports showing summary measures of the data across the sample and will be the only committee to receive closed reports displaying summary measures of the data split by treatment group.

### Plans for communicating important protocol amendments to relevant parties (e.g. trial participants, ethical committees) {25}

Any subsequent amendments to the protocol will be submitted to the TSC/DMEC, the Funder, and the REC and Regulatory Authorities for approval. They will be communicated to trial registries, journals, and trial participants, as appropriate. The PI will submit a final report at conclusion of the trial to the Funder, the REC, and the Sponsor.

### Dissemination plans {31a}

We anticipate the key beneficiaries of our research to be people with psychosis who are affected by past trauma; academics; clinicians and mental health service providers; and NICE guideline development group. It is intended that the results of the study will be reported and disseminated at international conferences and in peer-reviewed scientific journals and will be made available to participants and clinical teams in an accessible format and on the study website. Trial findings will also be accessible in print and digital media and presented at stakeholder’s events. One of the key outputs of this study will be the publication of the final, detailed therapy manual, which will include specific guidelines for the delivery of therapy and operational guidelines for its application, including case examples. A key aspect of the long-term dissemination will be through settings associated with health-care provision, such as presentations and workshops for CBT practitioners and health-care managers.

## Discussion

Improving access to psychological therapies and the implementation of trauma-informed care are key issues for NHS services. The proposed therapy, if acceptable, will provide a new, integrated psychological therapy for people with complex mental health problems whose needs are often not met by mental health services. The outcomes of this study, if positive, will be immediately useful to patients, clinicians, and clinical services. The proposed intervention has the potential to provide significant benefits to the substantial number of people who are affected by past trauma in terms of reductions in distressing post-traumatic stress, psychosis, and other symptoms. If found to be acceptable and safe, the therapy could overcome obstacles in therapy delivery in clinical practice such as clinicians’ concerns about the potential of trauma-focused therapy to exacerbate psychosis symptoms, and lack of confidence or competence in effectively treating trauma-related problems in psychosis populations [105, 106]. Importantly, it has the potential to be cost-effective to service providers through reduced need for participants to receive support from other health and social care services.

As well as meeting unmet needs for populations within secondary care mental health services, the therapy could also address the gap in provision that exists between primary care and secondary care. Many people with psychosis are denied entry into secondary care services, despite the presence of distressing symptoms, because they do not meet the increasingly high threshold set by Community Mental Health Teams and Early Intervention for Psychosis services, which are often based on complexity of social needs, lack of general functioning and immediate risk of hospitalisation, rather than presenting distress. Therefore, individuals assessed as functioning ‘well enough’, or not currently presenting with sufficient acuity or in crisis, are referred back to primary care, but their psychosis diagnosis excludes them from primary care psychological therapy services. This trial would potentially provide an evidence-based intervention to meet the needs of this currently neglected population.

The availability of a detailed therapy manual will enable the therapy to be applied by CBT-trained therapists throughout the NHS, in both primary and secondary care settings and elsewhere. There are current plans for a significant expansion of training in CBTp nationally by NHS-England (NHS-E), which will be timely for maximum impact of the manual, as it can be embedded within the curriculum of the national training programs.

The main barrier to providing immediate patient benefit is likely to be a lack of resources for the implementation of the therapy. However, providing parity of care for mental health is an ongoing, pan-party government agenda. The development of psychological interventions for psychosis, specifically, is a current NHS priority, with recent investments by NHS-E in Improving Access to Psychological Therapies – Severe Mental Illness (IAPT-SMI) demonstration sites [107], and the implementation of national standards for the access and waiting times for psychological therapies in Early Interventions for Psychosis services [108]. The remit of IAPT-SMI has since expanded (now renamed Psychological Therapies for SMI), with training plans contributing to a national agenda for increasing the workforce. The recent inclusion in NICE guidelines of the necessity to assess and treat trauma symptoms in people with psychosis in Early Interventions for Psychosis services is an indication that this topic is timely and will remain highly relevant to the needs of the NHS. Pathways to specialist trauma therapy are also integral to the provision of trauma-informed care, which is recommended in the NHS Long Term Plan as the model for community services in adults with severe mental health problems [109]. A failure to treat trauma sequelae in psychosis is itself costly to patients, their families, and the NHS. Should the therapy prove cost-effective, it will provide evidence to Clinical Commissioning Groups that investing in this treatment will save money in the long run.

## Trial status

This protocol is Version 3.06 (DATE: 21.01.2022). Recruitment is planned to start in October 2020 and to last 22 months until July 2022. Follow-up data collection is planned to be completed in August 2024.

## Supplementary Information

**Additional file 1: Appendix A**. STAR Consent Form

## Abbreviations

AD-SUS: Adult Service Use Schedule
AEs: Adverse events
ASSIST: Alcohol, Smoking and Substance Involvement Screening Test
CAPS-5: Clinician-Administered PTSD Scale for DSM-5
CBT: Cognitive behaviour therapy
CBTp: Cognitive behaviour therapy for psychosis
CDSS: Calgary Depression Scale
CHOICE: CHoice of Outcome In Cbt for psychoses
CI: Confidence interval
CONSORT: Consolidated Standards of Reporting Trials
CONSORT-SPI: Consolidated Standards of Reporting Trials – Social and Psychological Interventions
CTS-R: Cognitive Therapy Scale – Revised
CTU: Clinical Trials Unit
DASS-21: Short form of the Depression Anxiety Stress Scales
DSO: Disturbances in Self-Organisation
DSPS: Dissociative Subtype of PTSD Scale
DMEC: Data Management and Ethics Committee
DSM-5: Diagnostic and Statistical Manual of Mental Disorders-5^th^ Edition
EDC: Electronic Data Capture
EQ-5D-3 L: EuroQol 3-dimensions
EQ-5D-5L: EuroQol 5-dimensions
ES: Effect size
GDPR: General Data Protection Regulation
GP: General Practitioner
GPTS-R: Revised Green et al Paranoid Thoughts Scale
HEIs: Higher Education Institutions
HTA: Health Technology Assessment
IAPT-SMI: Improving Access to Psychological Therapies for Severe Mental Illness
ICCs: Intraclass correlation coefficients
ICD-10: International Classification of Diseases 10th Edition
ICD-11: International Classification of Diseases 11th Edition
IT: Information Technology
ITQ: International Trauma Questionnaire
ITT: Intention to treat
KCL: King’s College London
KCTU: King’s Clinical Trials Unit
m: Months
*N*: Number
NHS: National Health Service
NHS-E: NHS-England
NICE: National Institute for Health and Care Excellence
NIHR: National Institute of Health Research
PCL-5: PTSD Checklist for DSM-5
PI: Principal Investigator
PICuP: Psychological Intervention Clinic for outpatients with Psychosis
PID: Personally Identifiable Data
PPI: Patient Public Involvement
PSFS: Paykel Suicidal Feelings Scale
PSP: Personal and Social Performance Scale
PSSRU: Personal Social Services Research Unit
PSYRATS: Psychosis Symptom Rating Scales
PTCI: Posttraumatic Cognitions Inventory
PTSD: Post-traumatic stress disorder
QALYs: Quality-adjusted life years
RCT: Randomised controlled trial
R&D: Research & Development
REC: Research Ethics Committee
ReQoL: Recovering Quality of Life
SAEs: Serious adverse events
SD: Standard deviation
SLaM: South London and Maudsley NHS Foundation Trust
SMI: Severe mental illness
SREs: Serious related events
TALE: Trauma And Life Events Checklist
TAU: Treatment as usual
TF-CBT: Trauma-Focused Cognitive Behaviour Therapy
TF-CBTp: Trauma-Focused Cognitive Behaviour Therapy for psychosis
TSC: Trial Steering Committee
UK: United Kingdom
USREs: Unexpected serious related events
WHO: World Health Organization
≥: equal to or above
