# Supplementary figures and images for: Multisite randomised controlled trial of trauma-focused cognitive behaviour therapy for psychosis to reduce post-traumatic stress symptoms in people with co-morbid post-traumatic stress disorder and psychosis, compared to treatment as usual: study protocol for the STAR (Study of Trauma And Recovery) trial
Source: Trials. 2022 May 23;23:429. doi: 10.1186/s13063-022-06215-x (PMC9125351; doi:10.1186/s13063-022-06215-x)

Appendix A: STAR Consent Form


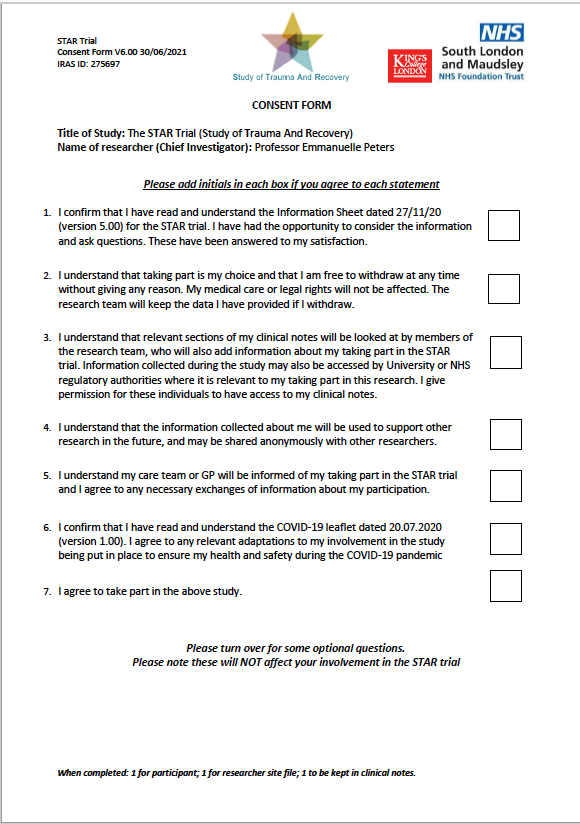


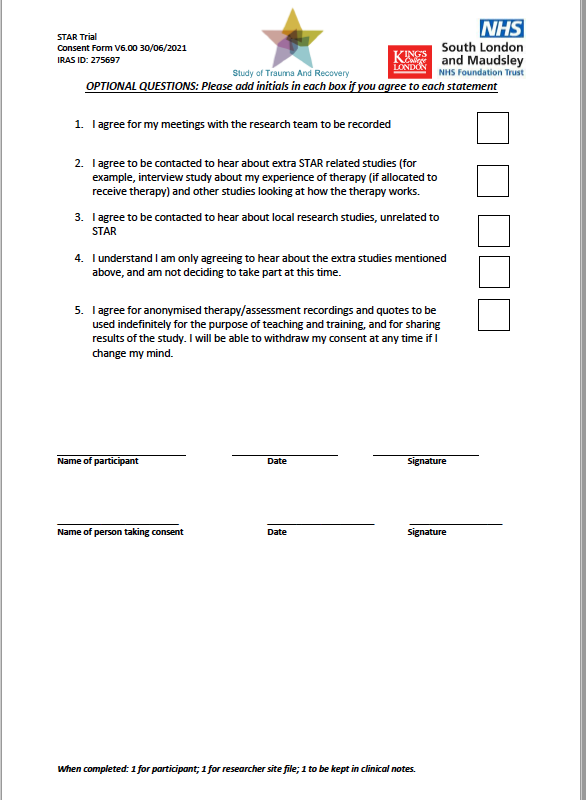

Supplement: Supplementary file 1 — Additional file 1: Appendix A. STAR Consent Form [file 13063_2022_6215_MOESM1_ESM.docx]
